# Measuring HIV Stigma at the Family Level: Psychometric Assessment of the Chinese Courtesy Stigma Scales (CCSSs)

**DOI:** 10.1371/journal.pone.0092855

**Published:** 2014-03-21

**Authors:** Hongjie Liu, Yongfang Xu, Yehuan Sun, Levent Dumenci

**Affiliations:** 1 Department of Epidemiology and Biostatistics, School of Public Health, University of Maryland, College Park, Maryland, United States of America; 2 Department of HIV/AIDS Control, Nanning Center for Disease Control and Prevention, Nanning, Guangxi, China; 3 Department of Epidemiology, Anhui Medical University, Hefei, Anhui, China; 4 Department of Social and Behavioral Health, Virginia Commonwealth University, Richmond, Virginia, United States of America; University of Stellenbosch, South Africa

## Abstract

Courtesy stigma is the stigmatization a person perceives or experiences due to their association with a stigmatized individual or group. Most HIV-related stigma scales have been developed for people living with HIV/AIDS (PLWHAs), but not for their HIV-uninfected family members. To date, few measurement scales have been designed to measure the degree of stigma among both PLWHAs and their HIV-uninfected family members at the family level. We developed a set of courtesy stigma scales and estimated their reliability and validity from 256 PLWHAs and 256 of their HIV-uninfected family members. Exploratory and confirmatory factor analyses were performed in two independent samples: a development sample (N = 216) and a validation sample (N = 296), respectively. Two factors (“public stigma” and “self-perceived stigma”) had high internal consistency reliability (Cronbach's alpha coefficient between 0.83–0.90) and good construct validity (standardized factor loading range: 0.37–0.95) in both samples. These findings document that the newly developed brief instrument is a psychometrically sound measure of HIV-related stigma among both PLWHAs and their HIV-uninfected family members.

## Introduction

Stigma continues to be a major barrier to the treatment-as-prevention strategy for HIV interventions [1], [2]. Despite ongoing efforts to reduce stigma among people affected by HIV/AIDS, its deleterious effects persist. Stigma does not only exist in people living with HIV/AIDS (PLWHAs), but their family members, relatives, neighbors, and communities with which they are affiliated [3]. Stigma, in general, has been defined as the result of the interactions of its components – labeling, stereotyping, separation, status loss, and discrimination [4]. With regard to the HIV/AIDS epidemic, HIV-related stigma refers to prejudice, discounting, discrediting, and discrimination directed at people who are infected or affected by HIV/AIDS [5], [6]. Previous studies have documented that a higher level of HIV-stigma was strongly associated with a higher level of depression and a low level of self-efficacy [7]–[10]. As reported in a longitudinal study conducted in South Africa, HIV-related stigma persists across time and mediates the relationship between HIV/AIDS orphanhood and psychological distress (anxiety and depression) [11].

As posited by Goffman, perceived or experienced stigma could be passed on to family members of those with the stigmatizing attribute and has been coined “courtesy stigma” [12]. Courtesy stigma refers to a person who perceives or experiences stigmatization due to their association with a person who bears the chastised attribute. Courtesy stigma causes feelings of social isolation, shame and fear, and introduces additional stressors to HIV-uninfected family members of PLWHAs. These stressors add extra burden to already overwhelmed families and lead to the breakdown of social support [13]. Several studies have shown that HIV uninfected caregivers often maintain silence about their relatives' condition out of fear of stigma and discrimination to the family unit [14]–[16]. Despite these studies citing evidence for courtesy stigma, few, according to two systematic reviews [6], [17], have empirically investigated and compared the degree of HIV-related stigma and its consequences between PLWHAs and their HIV-uninfected family members at the family level.

The Chinese culture may foster stigma [15]. Different from the western countries where individualist culture prevails, the Chinese culture is more collectivist. Individuals with collectivist cultures tend to maintain respect, family dignity, and social status in the social structure in which they live [18]. As HIV infection is contagious and associated with stigmatizing behaviors (e.g. casual sex and drug use), people living with HIV/AIDS are usually devalued by a collectivist society because family or group value is considered to be damaged by these individuals. Because the cultural imperative of familial responsibility and social harmony, not only the HIV infected person, but also their family members are highly stigmatized in China [19], [20]. Due to the feeling of “deservingness”, HIV stigma may also be associated with transmission routes. That is, those who contract HIV through culturally-unaccepted behaviors (e.g., commercial sex) or practices (e.g., drug use) may experience greater stigma than those who contract it unintentionally, e.g., blood donors who were infected through HIV-contaminated blood collection equipment.

Despite the presence of psychometrically sound measures of HIV-related stigma among a variety of populations, including PLWHAs [21], [22], healthcare providers of PLWHAs [23], [24], HIV-affected youth and children [25], [26], men who have sex with men [27], and general populations in South Africa [28] and Yemen [29], few studies have investigated HIV-related stigma at the affected family level. This deficiency may reflect the absence of a validated scale to measure HIV stigma in both PLWHAs and their HIV-uninfected family members.Therefore, we designed a set of brief measurement scales to assess HIV-related stigma perceived by PLWHAs and courtesy stigma perceived by HIV-uninfected family members of PLWHAs. The individual indicators in this set broadly covered the major domains of stigma: stereotyping, separation, prejudice, discounting, status loss, and discrimination directed at individuals who are either infected and/or affected by HIV/AIDS. The primary objective of this study was to assess psychometric properties of the Chinese Courtesy Stigma Scales (CCSSs) in the Chinese population

## Materials and Methods

The study protocol was approved by the Institutional Review Boards of Virginia Commonwealth University, the Guangxi Center for Disease Control and Prevention and the Anhui Medical University Institute of Biomedicine. In accordance with the approved protocol, written informed consent was obtained from all study participants prior to data collection.

### Study site and participants

We conducted two cross-sectional studies among HIV affected families in Anhui and Guangxi, China in 2008 and 2010 [30]. Interviewers were trained in questionnaire administration, developing rapport with participants and issues of confidentiality.

The first cross-sectional study was conducted among PLWHAs and their family members in a rural area in Anhui province. The majority of farmers were infected with HIV through commercial plasma donations that occurred in the early-to-mid 1990s [31]. Eligible subjects included PLWHAs and one of their family members who were at least 18 years old. PLWHAs who could not participate in an interview due to poor health conditions were excluded from this study. Based on the local HIV surveillance data, we first selected villages with high HIV prevalence, and then listed all HIV-infected families. In each village, all HIV infected families were invited to participate in the study. In each family, one HIV-infected family member and one HIV-uninfected family member who was either a spouse or parent (depending on the marital status of the participant) were invited to receive face-to-face interviews. Interviews were conducted in a private room of the participant's home with only the interviewer and participant present.

The second cross-sectional study was conducted in Nanning, Guangxi. The study methodology has been described elsewhere [30]. Briefly, this study was conducted among PLWHAs and their caregivers at the dyadic level. The province had the second highest rate of HIV infection in China. The major transmission routes of HIV is through heroin injection and risky sexual behavior [32]. We selected three study sites in the city that provided HIV care and treatment services for the majority of PLWHAs in that city: an infectious disease hospital that was designated to provide care and treatment for PLWHAs, a methadone maintenance treatment clinic run by the Nanning Center for Disease and Control, and a health-care center run by PLWHA volunteers. Eligibility criteria included PLWHAs who were at least 18 years old and able to receive a face-to-face interview. After obtaining a participant's written informed consent, a trained interviewer administered a face-to-face interview in a private room. Caregivers were eligible if they met the following criteria: (a) primary caregivers to the corresponding PLWHAs, (b) age 18 or older, and (c) HIV negative. After their eligibility was confirmed, caregivers received the same face-to-face interviews as did the PLWHAs.

The development sample was taken from Anhui where 108 HIV-infected individuals and 108 of their HIV-uninfected family members participated in the interview. At this site, 118 HIV affected families were invited to participated, 10 families were excluded as one or two family members declined to participate or did not provide information regarding stigma. The major mode of HIV transmission at the study site was unsafe commercial blood donation practices. The validation sample was taken from Guangxi where 148 HIV-infected individuals and 148 of their HIV-uninfected family members participated in the interview. Of 170 HIV dyads invited to participate in this study, 20 dyads refused, and 2 dyads did not provide information about their perceived stigma and were excluded. The primary mode of HIV transmission in this region was injection drug use (IDU).

### Measures

Instruments were initially drafted in English and then translated into Chinese by research members who were fluent in both languages. The Chinese version of the items was then distributed to research team members who reviewed and modified the wording to make it appropriate for the Chinese context. The development of courtesy stigma scales was based on our previous studies [27], [33]–[35]. The CCSSs was designed to measure two facets of stigma: public and self-perceived stigma [36], [37]. Public stigma is the attitudes or reactions that the general population has toward people who have a particular undesirable attribute, such as HIV infection. Self-perceived stigma, on the other hand, refers to the fear of societal attitudes and potential discrimination perceived by people who have the undesirable attribute. To measure public stigma, we developed 13 items with a 4-point ordinal response format (*strongly agree, agree, disagree, strongly disagree*). Examples of the items included: “*No one would be willing to take care of their children when HIV infected people die from AIDS.*” and “*People seldom buy food from HIV-infected individuals or their family*”. To measure self-perceived stigma, we developed 9 items with a 4-point ordinal response format (*a lot, some, a little, none*). Examples of items included: “*Because of my family member's HIV status, I feel shame and self-blame*” and “*Because of my family member's HIV status, I feel that children are kept away from me by their parents*”.

In addition to the 22-item courtesy stigma scales, we administered depression and self-efficacy scales to provide concurrent validity evidence for the newly developed instrument. *CES-D (Center for Epidemiologic Studies Depression Scale)* is a 10-item scale designed to measure depressive symptoms experienced in the past week [38]. Response format ranges from 1 to 4 as rarely or none of the time (less than 1 day), some or a little of the time (1–2 days), occasionally or a moderate amount of the time (3–4 days), and most or all of the time (5–7 days). In the development sample, Cronbach's alpha coefficient was 0.80 in the HIV-uninfected individuals and 0.78 in the HIV-infected individuals. In the validation sample, Cronbach's alpha coefficient was 0.85 in the HIV-uninfected individuals and 0.82 in the HIV-infected individuals. The CES-D total score was obtained by adding responses to all 10 items (range: 1–40). High scores indicate high frequency of depressive symptom episodes.

The general self-efficacy scale was used to measure a general sense of perceived self-efficacy [39]. The scale consists of 10 items, e.g., “I can always manage to solve difficult problems if I try hard enough” and “I can solve most problems if I invest the necessary effort.” Participants responded on a four-point item response scale ranging from “not at all true (1)” to “exactly true (4).” In the development sample, Cronbach's alpha coefficient was 0.86 in the HIV-uninfected individuals and 0.90 in the HIV-infected individuals. In the validation sample, Cronbach's alpha coefficient was 0.84 in the HIV-uninfected individuals and 0.85 in the HIV-infected individuals. High scale scores indicate high levels of self-efficacy.

### Data Analyses

Exploratory factor analysis (EFA) with oblique rotation was performed to assess the dimensionality of the CCSS items that underlie the sample data and to identify the measurement structure of the test. Two-factor solutions were inspected separately for PLWHAs and their HIV-uninfected relatives. Scree plots, factor patterns and factor structure coefficients, the number of items with high loadings on one factor and low on the remaining factors, and the theoretical meaningfulness of identified factors were considered in deciding the measurement model for the courtesy scales. The number of eigenvalues preceding the elbow in scree plots was retained for rotation; remaining eigenvalues were deemed unimportant and were subsequently dropped. The cumulative proportion of variance was obtained from successive factor solutions. The EFA procedures were carried out in MPlus version 7.1 using an asymptotically distribution-free estimator, i.e., weighted least squares estimator with robust standard errors and mean- and variance-adjusted chi-square values (WLSMV), given the ordered categorical nature of item distributions and an oblique rotation (i.e., geomax).

Confirmatory factor analysis (CFA) was used in the validation sample to replicate the measurement model suggested by EFA in the development sample. Goodness of fit was assessed using chi-square test of exact fit (non-significant p-value as a good fit), root mean square errors of approximation (RMSEA; ≤0.08 as a good fit), Comparative Fit Index (CFI; ≥0.90) and Tucker Lewis Index (TLI; ≥0.90) [40]. Because each fit index has its own strengths and weaknesses, meaningfulness of parameter estimates were also taken into account in determining the model fit. Cronbach's alpha coefficient was used to assess the internal consistency of scale scores. A composite score was also calculated for each of the scales. CFA was carried out in Mplus (version 7.11) [41] using the WLSMV estimator.

Concurrent validity was assessed by evaluating associations between the courtesy stigma scales and depression and self-efficacy scores. Based on previous research [7]–[10], we expected that the HIV-related stigma scales were positively associated with the depression scores but negatively associated with self-efficacy scores in the two independent samples. Additionally, we expected that the HIV public stigma score was positively correlated with the self-perceived stigma score. Pearson correlation coefficients (r) were estimated.

## Results

### Development and exploratory analyses

The development sample consisted of 216 subjects (108 PLWHAs and 108 HIV-uninfected family members) who participated in the interview. The descriptive statistics are presented separately by HIV status in Table 1. The HIV positive sample had a mean age of 45.3 years (Standard deviation (SD)  = 8.6, range: 18–69 years old).The majority of the HIV positive sample was female (57.4%), had a primary school education or no education (82.5%), were farmers (68.5%), and married (84.3%) at the time of interview. The HIV negative sample had a mean age of 37.1 years (SD  = 14.3, range: 18–75 years old). Sixty percent were female, 66.7% had a primary school education or no education, 51.9% were farmers, and 83.3% were married at the time of interview.

**Table 1:** Social demographic characteristics of the two samples.

	HIV–infected sample (N = 108)	HIV-uninfected sample (N = 108)
**Development Sample**		
Gender		
Male	46 (42.6%)	43 (39.8%)
Female	62 (57.4%)	65 (60.2%)
Age (Mean, SD)	45.3 (8.6)	37.1 (14.3)
Education Level		
No School	56 (51.9%)	25 (23.2%)
Primary School	33 (30.6%)	47 (43.5%)
Middle School or above	19 (17.6%)	36 (33.3%)
Occupation		
Farmer	74 (68.5%)	56 (51.9%)
others*	34 (31.5%)	52 (48.1%)
Marriage Status		
Married or Remarried	91 (84.3%)	90 (83.3%)
Unmarried, Widow/er, divorced or Separated	17 (15.7%)	18 (16.7%)
**Validation Sample**	HIV-infected sample (N = 148)	HIV–uninfected sample (N = 148)
Gender		
Male	103 (69.6%)	58 (39.2%)
Female	45 (30.4%)	90 (60.8%)
Age (Mean, SD)	40.7 (11.7)	37.5 (11.2)
Education Level		
No School	7 (4.7%)	4 (2.7%)
Primary School	31 (21.0%)	30 (20.3%)
Middle School or above	110 (74.3%)	114 (77.0%)
Occupation		
Farmer	40 (27.0%)	36 (24.3%)
Others	108 (73.0%)	112 (75.7%)
Marriage Status		
Married or Remarried	108 (73.0%)	123 (83.1%)
Unmarried, Widow/er, Divorced or Separated	40 (27.0%)	25 (16.9%)

* “Others” include: migrant laborer, self-employed, student, driver or others.

**Figure 1:**
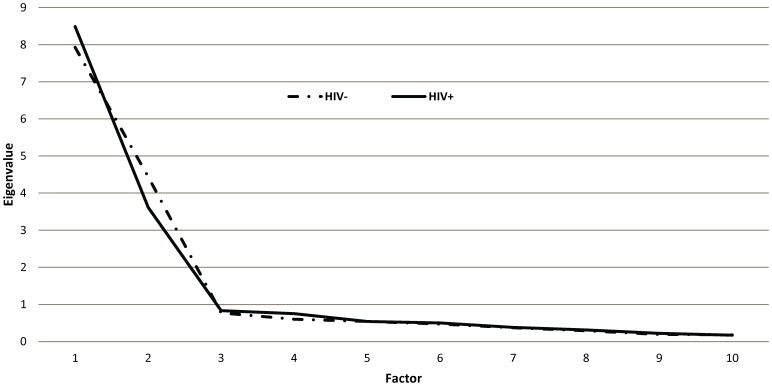
Scree test for eigenvalues in the development sample.

The EFA was conducted on the development sample. The scree plots obtained separately from HIV-infected and-uninfected samples suggested a two-factor solution (see Figure 1), as hypothesized, involving public HIV stigma and self-perceived HIV stigma. Two factors explained 83% of variance in item responses in the HIV-uninfected sample and 81% in the HIV-infected sample. In both samples, each item loaded high on one factor (range: 0.47–0.86) and low on the other (range: −0.15–0.23) producing a robust simple structure. These results provided further evidence that the correlated two-factor solution was most appropriate. Considering prior knowledge on the dimensions of stigma, the results suggested that two factors sufficiently explain the correlations among item responses in the development sample. Two factors were labelled as “public HIV stigma” and “self-perceived HIV Stigma.” The internal consistency of both scale scores estimated from the Cronbach's alpha coefficient were as follows: 0.93 for the public HIV stigma scale in the HIV-infected sample, 0.93 for the public HIV stigma scale in the HIV-uninfected sample, 0.91 for the self-perceived HIV stigma scale in the HIV-infected sample, and 0.92 for the self-perceived HIV stigma scale in the HIV-uninfected sample (Table 2).

**Table 2:** Concurrent validity analysis of courtesy stigma.

				Correlation	
Factors	Cronbach's alpha	Mean	SD†	Public Stigma	Self-perceived Stigma
**Development sample**					
**Among HIV-infected sample**					
HIV public stigma	0.93	13.7	6.8	1.00	0.36**
HIV Self-perceived Stigma	0.91	8.5	6.9	0.36**	1.00
Depression	0.78	21.4	6.4	0.01	0.30**
Self-efficacy	0.90	27.3	4.9	−0.05	−0.16
**Among HIV-uninfected sample**					
HIV public stigma	0.93	11.5	6.9	1.00	0.23**
HIV Self-perceived Stigma	0.92	5.2	6.1	0.23**	1.00
Depression	0.80	18.0	5.9	0.20**	0.45**
Self-efficacy	0.86	28.5	4.3	−0.14	−0.04
**Validation sample**					
**Among HIV-infected sample**					
HIV public stigma	0.87	18.0	4.4	1.00	0.41**
HIV Self-perceived Stigma	0.83	8.5	5.1	0.41**	1.00
Depression	0.82	21.3	6.4	0.28**	0.43**
Self-efficacy	0.85	24.9	3.4	−0.09	−0.13
**Among HIV-uninfected sample**					
HIV public stigma	0.90	16.2	4.8	1.00	0.18*
HIV Self-perceived Stigma	0.88	5.7	5.0	0.18*	1.00
Depression	0.85	17.7	6.3	0.17*	0.62**
Self-efficacy	0.84	26.6	3.3	−0.02	−0.25**

† =  Standard Deviation *p≤0.05 **p≤0.01.

To assess their concurrent validity, we estimated the correlations of the stigma scales with depression and self-efficacy in two independent samples of the HIV-infected individuals and HIV-uninfected individuals. Table 2 illustrates the means, standard deviations and correlations of the scale scores. In the development sample, the public stigma was significantly correlated with self-perceived stigma (r = 0.36, p<0.01), but not with depression (r = 0.01; p = 0.90) or self-efficacy (r = −0.05; p = 0.64) in the HIV positive sample. In the HIV-uninfected sample, public stigma was significantly correlated with self-perceived stigma (r = 0.23; p<0.01) and depression (r = 0.20; p<0.01), but not with self-efficacy (r = −0.14; p = 0.15). HIV self-perceived stigma was significantly correlated with depression (r = 0.30; p<0.01), but not with self-efficacy (r = −0.16; p = 0.10) in the HIV infected sample. In the HIV-uninfected sample, self-perceived stigma was significantly correlated with depression (r = 0.45; p<0.01), not with self-efficacy (r = −0.04; p = 0.68).

### Confirmatory and validation analyses

The validation sample consisted of 296 subjects (148 PLWHAs and 148 HIV-uninfected family members). The HIV-infected sample had a mean age of 40.7 years (SD: 11.7, range: 20-80 years old). One third (30.4%) of the HIV-infected sample was female, 25.7% had a primary school education or no education, 27% were farmers, and 73% were married at the time of interview. The HIV-uninfected sample had a mean age of 37.5 years (SD = 11.2, range: 18–84 years old). Sixty-one percent were female, 23% had a primary school education or no education, 24.3% were farmers, and 83.1% were married at the time of interview.

To further validate the findings of the development sample from the EFA, we used confirmatory factor analysis (CFA) on the validation sample. Specifically, we fit the correlated two-factor model separately for the HIV-infected and HIV-uninfected samples. The CFA model specifications relied solely on the EFA results described above in order to cross-validate the measurement structure of stigma scales in two independent samples. Whereas the chi-square test of exact fit and RMSEA did not indicate good model fit, the CFI and TLI indices supported the model in both the HIV-infected sample (Chi-square  = 661.6, df  = 208, p<0.01; WRMR  = 1.73; CFI = 0.90; TLI = 0.90) and HIV-uninfected sample (Chi-square  = 651.6, df  = 208, p<0.01; WRMR  = 1.83; CFI = 0.92; TLI = 0.91). The standardized factor loading in public stigma was between 0.40–0.93 in HIV-infected sample and 0.62–0.93 in HIV-uninfected sample. The factor loading in self-perceived stigma was between 0.40–0.93 in HIV-infected sample and 0.37–0.95 in HIV-uninfected sample (Table 3). The internal consistency of scale scores estimated from the Cronbach's alpha coefficient were high: 0.87 for the public HIV stigma scale in the HIV-infected sample, 0.90 for the public HIV stigma scale in the HIV-uninfected sample, 0.83 for the self-perceived HIV stigma scale in the HIV-infected sample, and 0.88 for the self-perceived HIV stigma scale in the HIV-uninfected sample (Table 2).

**Table 3:** Standardized factor loading estimates from confirmatory factor analyses (the validation sample).

	HIV-infected sample	HIV-uninfected sample
Public HIV-related stigma		
1. HIV infected people should be ostracized by their spouse and family members	0.48	0.81
2. HIV infected people would lose their friends if they knew their HIV status.	0.80	0.88
3. HIV infected people should be forced to leave their villages.	0.53	0.69
4. HIV infected people's family would not care for them.	0.40	0.74
5. No one would be willing to take care of their children when HIV infected people die from AIDS.	0.50	0.76
6. Children should not go to school because their parents are infected with HIV.	0.57	0.93
7. HIV infected people should not have the same rights to education and employment as others.	0.57	0.66
8. People would not be willing to socialize with HIV/AIDS patients.	0.90	0.73
9. People seldom buy food or vegetables from HIV/AIDS patients or their family.	0.91	0.78
10. People think HIV infection is a punishment for their bad behaivor.	0.65	0.62
11. People would not like to marry HIV infected people.	0.84	0.73
12. Students would not like to play with HIV infected people's children.	0.93	0.68
13. Parents would keep their children away from HIV infected people and their family.	0.83	0.87
Self-perceived HIV- Stigma		
For HIV negative subject/For HIV positive subject		
1. Because of my family member's HIV status, I feel estranged by people around me/Because of my HIV status, I feel estranged by people around me.	0.92	0.86
2. Because of my family member's HIV status, I feel blamed by people around me/Because of my HIV status, I feel blamed by people around me.	0.93	0.80
3. Because of my family member's HIV status, I feel shame and self-blame/Because of my HIV status, I feel shame and self-blame.	0.72	0.85
4. Because my family member's HIV status, I feel it is very hard for my family members to get married/Because my HIV status, I feel it is very hard for my family members to get married.	0.77	0.90
5. Because of my family member's HIV status, I feel it is uneasy to get along with people around me/Because of my HIV status, I feel it is uneasy to get along with people around me.	0.69	0.95
6. Because of my family member's HIV status, I feel I am inferior to others in many respects/Because of my HIV status, I feel I am inferior to others in many respects.	0.75	0.95
7. Because of my family member's HIV status, I feel people will no longer see my strong points/Because of my HIV status, I feel people will no longer see my strong points.	0.76	0.83
8. Because of my family member's HIV status, I feel that children are kept away from me by their parents/Because of my HIV status, I feel that children are kept away from me by their parents.	0.40	0.37
9. Because of my family member's HIV status, I feel my family members cannot have the same rights to education and employment as others/Because of my HIV status, I feel my family members cannot have the same rights to education and employment as others.	0.46	0.64

The results of the concurrent validity analyses indicated the expected correlations among HIV public stigma, self-perceived stigma, depression, and self-efficacy. Specifically, public stigma was significantly correlated with self-perceived stigma (r = 0.41, p<0.01) and depression (r = 0.28; p<0.01), but not with self-efficacy (r = −0.09; p = 0.26) among in the HIV positive sample. In the HIV-uninfected sample, public stigma was significantly correlated with self-perceived stigma (r = 0.18; p = 0.03) and depression (r = 0.17; p = 0.04), but not with self-efficacy (r = −0.02; p = 0.84). HIV self-perceived stigma was significantly correlated with depression (r = 0.43; p<0.01), but not with self-efficacy (r = −0.13; p = 0.13) in the HIV-infected sample. In the HIV-uninfected sample, self-perceived stigma was significantly correlated with depression (r = 0.62; p<0.01) and self-efficacy (r = −0.25; p<0.01) (Table 2).

## Discussion

The findings of this study demonstrate that the CCSSs can reliably capture courtesy stigma among HIV-uninfected family members of PLWHAs. Because the same set of stigma scales can be used in both PLWHAs and their family members, comparisons of the two types of stigma can be directly performed at the dyadic or family level. Results from factor analyses showed that these scales shared the same factor structure in both HIV-infected and HIV-uninfected samples, indicating consistency across two different populations. High Cronbach's alpha coefficients supported the internal consistency and reliability of the HIV stigma scales. The alpha of 0.90 for public stigma and 0.88 for self-perceived stigma among the HIV-uninfected family members provides confidence that the CCSS reliably measures two dimensions of courtesy stigma.

Concurrent validity of the CCSS scales was supported by significant correlations among the stigma scale with depression and self-efficacy, which previous studies have shown to be closely related to stigma [7]–[10]. Significant correlations between self-perceived stigma and public stigma, depression and public stigma, and depression and self-perceived stigma scales among both the HIV-uninfected and HIV-infected groups supported the concurrent validity of the CCSS scale scores. However, only among the HIV negative sample, did we detect a significant correlation between self-perceived stigma and self-efficacy scales. Low to moderate correlations between public HIV-related stigma and self-perceived HIV-stigma scales supported the discriminant validity of the CCSS.

While previous studies have assessed psychometric characteristics of HIV-related stigma scales in particular populations, few studies have empirically assessed an instrument that could be used with equal accuracy among PLWHA and their HIV-uninfected adult family members. Our findings document sound psychometric properties of the CCSS scales to measure HIV courtesy stigma among family members of HIV-infected individuals as well as among PLWHA themselves. The psychometric properties were very robust in the two different study populations with different HIV transmission modes (injection drug use vs. commercial blood donation) and across different investigation periods (2008 vs. 2010). The CCSSs have advantages over existing instruments in that it is brief, easy to administer, and applicable to diverse populations.

Despite the many strengths of this study, some limitations should be noted. While EFA/CFA strongly supported the correlated two-factor structure of the CCSSs in two samples, fit indices from CFA in two independent samples provided mixed support for the measurement structure. Future studies with larger sample sizes are needed to further test the proposed measurement model for the CCSSs in independent samples. While our results show that there is a reliable scale for measuring courtesy stigma among HIV-uninfected people in the study population, there may be some limitations of the generalizability to different populations, especially to those in individualistic Western cultures. As the major HIV transmission routes in this study were blood donation and heroin injection, these measurement scales may not be appropriately used among HIV-infected people who contract HIV via other transmission modes (e.g., vertical transmission or sexual transmission).

Our study provides a valuable tool for measuring HIV-related stigma for PLWHAs and courtesy stigma for their HIV negative family members in China and other countries with similar settings. The findings of this study may provide insight into new ways to improve current HIV-related stigma scales, particularly those targeting HIV-uninfected family members who perceive or experience courtesy stigma. The new scales can be used in the development and implementation of family-focused public health interventions to address stigma across HIV-affected families that extend beyond the PLWHAs.
